# Editorial: Non-invasive brain stimulation in psychiatric disorders: from bench to bedside, volume II

**DOI:** 10.3389/fpsyt.2023.1317954

**Published:** 2023-10-27

**Authors:** Chih-Wei Hsu, Po-Han Chou, Shao-Cheng Wang, Kuan-Pin Su

**Affiliations:** ^1^Department of Psychiatry, Kaohsiung Chang Gung Memorial Hospital and Chang Gung University College of Medicine, Kaohsiung, Taiwan; ^2^Department of Psychiatry, China Medical University Hsinchu Hospital, China Medical University, Hsinchu, Taiwan; ^3^Dr. Chou's Mental Health Clinic, Hsinchu, Taiwan; ^4^Department of Psychiatry, Taoyuan General Hospital, Ministry of Health and Welfare, Taoyuan, Taiwan; ^5^Department of Mental Health, Johns Hopkins Bloomberg School of Public Health, Baltimore, MD, United States; ^6^Mind-Body Interface Laboratory (MBI-Lab), China Medical University Hospital, Taichung, Taiwan; ^7^College of Medicine, China Medical University, Taichung, Taiwan; ^8^An-Nan Hospital, China Medical University, Tainan, Taiwan

**Keywords:** transcranial alternating current stimulation (tACS), theta burst stimulation (TBS), transcranial electrical stimulation (tES), transcranial direct current stimulation (tDCS), transcranial magnetic stimulation (TMS)

In the treatment of psychiatric disorders, pharmacological interventions sometimes fail to achieve adequate therapeutic effects. This has led to the emergence of numerous non-pharmacological treatments such as non-invasive brain stimulation (NIBS) (1). NIBS broadly covers technologies such as repetitive transcranial magnetic stimulation (rTMS), transcranial electrical stimulation, vagus nerve stimulation, cranial electrical stimulation, transcranial near-infrared radiation, and even electroconvulsive therapy (ECT), and has gained considerable attention in clinical and research fields in recent years. Specifically, rTMS was approved by the U.S. Food and Drug Administration for the treatment of major depressive disorder in 2008 (2) and for the treatment of obsessive-compulsive disorder in 2018 (3). Moreover, NIBS was demonstrated in many studies investigating having therapeutic potential in other psychiatric disorders, such as schizophrenia (4), methamphetamine use disorder (5), Tourette syndrome (neurodevelopmental disorders) (6), and Alzheimer's disease (neurodegenerative disorders) (7). Given the proven therapeutic utility of NIBS in a variety of psychiatric disorders, a closer look at the factors that may modulate the therapeutic effectiveness of NIBS is warranted. These may include predictors of treatment outcome, or potential enhancing effects when taken with food or drugs. Furthermore, whether objective measurement tools can verify the success of NIBS intervention is also an important area of research. The current Research Topic consists of four articles that provide a perspective on these key considerations.

The biological background of patient may influence therapeutic outcomes. A review by Hanlon and McCalley suggests that being female could be a potential factor that makes one more sensitive to the therapeutic effects of rTMS. The authors offer three potential biological explanations for this: (1) shorter distance from the brain to the scalp, (2) greater gray matter density and gyrification, and (3) high levels of estradiol.

Interactions of NIBS with certain medications or dietary elements may enhance or inhibit therapeutic effects. For example, cycloserine, an N-methyl-D-aspartate partial agonist, supports its role as a potentiator of NIBS-induced motor cortical excitability (8). However, chronic caffeine use may be an inhibitor of NIBS-induced motor cortical excitability. The study by Vigne et al. which included 20 healthy participants, found that chronic caffeine consumption blunts rTMS-induced plasticity even when cycloserine is administered.

Objective measures related to NIBS efficacy can serve as validation or prediction instruments for treatment effectiveness. This Issue included two studies focusing on neuroimaging changes before and after treatment. The first study by Kroll et al. recruited 14 patients with depression who underwent ECT and used positron tomography to assess changes in brain A1 adenosine receptor availability before and after treatment (an average of 6 days). The results indicated no correlation between changes in clinical outcome parameters and regional A1 adenosine receptor availability. The second study by Chou et al. enrolled 26 patients with depression to undergo 20 sessions of rTMS treatment, employing near-infrared spectroscopy (NIRS) to evaluate pre-and post-treatment changes in frontal lobe activity (average 28 days). The study found that increased frontal lobe activity was associated with improvements in depressive symptoms in responders after 20 rTMS sessions. Furthermore, they found increases in frontal lobe activity after 10 rTMS sessions were also associated with symptom improvement after 20 rTMS sessions. However, these correlations were not observed among non-responders. In addition to potential treatment biomarker for schizophrenia (9), Chou et al.'s finding indicated that NIRS could be a potential biomarker in monitoring therapeutic effects of rTMS in the treatment of depression.

In summary, the articles collected in this issue investigate biological and nutritional factors that may modulate the therapeutic effects of NIBS, and explore the promise of neuroimaging as an objective measurement tool for establishing biomarkers of NIBS efficacy. We are optimistic that this Research Topic will contribute to further understanding of this complex and multidimensional field. Our deepest gratitude goes to the various research groups that have made scientific discoveries on this topic. Additionally, we would like to thank the reviewers who generously contributed their time and expertise to improve the quality of each study.

## Author contributions

C-WH: Writing—original draft. P-HC: Writing—review & editing. S-CW: Writing—review & editing. K-PS: Writing—review & editing.
